# Does a walk-through video help the parser down the garden-path? A visually enhanced self-paced reading study in Dutch

**DOI:** 10.3389/fpsyg.2022.1009265

**Published:** 2022-12-21

**Authors:** Sara Shoghi, Seçkin Arslan, Roelien Bastiaanse, Srdan Popov

**Affiliations:** ^1^International Doctorate in Experimental Approaches to Language and Brain (IDEALAB), University of Groningen, Netherlands/University of Newcastle, United Kingdom/University of Potsdam, Germany and Macquarie University, Sydney, NSW, Australia; ^2^Center for Language and Cognition Groningen (CLCG), University of Groningen, Groningen, Netherlands; ^3^CNRS, BCL, Université Côte d’Azur, Nice, France; ^4^Department of Neurosurgery, University Medical Center Groningen, Groningen, Netherlands

**Keywords:** syntactic ambiguity resolution, self-paced reading, visual context, sentence processing, PP attachment, written language comprehension

## Abstract

The human language processing mechanism assigns a structure to the incoming materials as they unfold. There is evidence that the parser prefers some attachment types over others; however, theories of sentence processing are still in dispute over the stage at which each source of information contributes to the parsing system. The present study aims to identify the nature of initial parsing decisions during sentence processing through manipulating attachment type and verbs’ argument structure. To this end, we designed a self-paced reading task using globally ambiguous constructions in Dutch. The structures included double locative prepositional phrases (PPs) where the first PP could attach both to the verb (high attachment) and the noun preceding it (low attachment). To disambiguate the structures, we presented a visual context in the form of short animation clips prior to each reading task. Furthermore, we manipulated the argument structure of the sentences using 2- and 3-argument verbs. The results showed that parsing decisions were influenced by contextual cues depending on the argument structure of the verb. That is, the visual context overcame the preference for high attachment only in the case of 2-argument verbs, while this preference persisted in structures including 3-argument verbs as represented by longer reading times for the low attachment interpretations. These findings can be taken as evidence that our language processing system actively integrates information from linguistic and non-linguistic sources from the initial stages of analysis to build up meaning. We discuss our findings in light of serial and parallel models of sentence processing.

## Introduction

Language is full of ambiguities, most of which are not even noticed when presented in an appropriate context. However, there are cases in which syntactic preferences disrupt language/sentence processing, for example, when the structural analysis or expectations of the parser mismatches the linguistic input. This is known as the *garden-path effect*, and it occurs when readers and/or listeners are led down an unintended or alternative interpretation of a sentence. For instance, in Bever’s (1970) famous garden-path sentence, *The horse raced past the barn fell*, the readers feel they fully understand the sentence until they reach the final word. The word *raced* is initially parsed as the predicate of the matrix verb with *the horse* as the subject. However, as soon as the verb *fell* appears, the readers realize they have been led down a garden path, and the phrase *raced past the barn* is, in fact, a reduced relative clause modifying the subject. The presence of the *garden-path effect* is taken to highlight the importance of syntax, suggesting that syntactic analysis is in progress as the sentence unfolds. Ever since this topic was introduced, there have been attempts to identify the nature of syntactic parsing preferences, which led to different theoretical explanations concerning how syntactic analysis occurs during sentence processing (e.g., Frazier, 1987; Sturt et al., 1999; Spivey et al., 2002; Pickering and Van Gompel, 2006; Ferreira and Tanenhaus, 2007). Most influential theoretical explanations include the *syntax-first* model (Frazier and Fodor, 1978) and the *constraint-based* accounts (Taraban and McClelland, 1988; Macdonald et al., 1994; McRae and Matsuki, 2013). However, these theoretical models are somewhat in disagreement over at which stage contextual information guides the parser through the garden path. In the current study, we investigate whether and how non-linguistic context influences syntactic parsing during word-by-word reading.

### Theoretical background

The *syntax-first* model, also known as the *two-stage* account, holds that during sentence comprehension, syntactic analysis is organized in a modular fashion (Rayner et al., 1983). According to this model, a dichotomy is present during sentence processing: First, only the syntactic information is used during the initial analysis, functioning as an encapsulated module. At a later stage, other sources of information (e.g., semantic, pragmatic, or contextual information) are activated to carry out the subsequent analyses (Frazier and Rayner, 1982; Ferreira and Clifton, 1986; Tanenhaus et al., 1995). When there is a mismatch between the initial syntactic analysis and the secondary non-syntactic analysis, reanalysis takes place. During reanalysis, the dispreferred (but correct) syntactic structure replaces the preferred one so that the correct sentence interpretation is obtained, and hence processing takes longer than usual. In sentence processing literature, this is also known as the *recovery mechanism*. Longer reading times as a result of the involvement of the *recovery mechanism* have often been taken as support for the *garden-path effect*. One of the most influential theories of sentence processing is the *garden-path theory* (Frazier, 1979, 1987) based on which syntactic preferences are driven by two universal principles of *minimal attachment* and *late closure*. According to *minimal attachment*, the parser builds the simplest structure in terms of syntactic relations, and based on *late closure*, if grammatically permissible, new items should be attached to the clause or phrase currently being processed. The *construal theory* (Carreiras and Clifton, 1993), which is the latest version of the *garden-path theory*, allows the application of *minimal attachment* and *late closure* principles only when the attachment constitutes a “primary relation” (i.e., a relation between arguments and their dependents). For other relations (i.e., adjuncts), there is initially no commitment to a particular syntactic alternative, but at a later stage, thematic and pragmatic information will give rise to a parsing preference.

An alternative explanation for parsing preferences is offered by the *constraint-based* model. The model assumes that multiple sources of information (constraints) such as general syntactic biases, word meaning, verb subcategorization information, contextual biases, and prosody of speech become available simultaneously or with only little delay and interact to make comprehension possible. This implies that different interpretations are activated in parallel, compete with one another over time, and are weighted probabilistically (Trueswell et al., 1993, 1994; McRae et al., 1998; McRae and Matsuki, 2013). The *constraint-based* model holds that multiple structural alternatives leading to different sentence interpretations are activated simultaneously. Depending on the degree to which each representation is activated, the “settling process” takes longer or shorter (Spivey et al., 2013). That is, if multiple constraints from semantic, syntactic, and contextual biases guide the correct interpretation, the settling process is shorter than when such biases favor the incorrect analysis. If the majority of the biases equally support both syntactic alternatives, the settling process becomes remarkably slow. When the majority of biases initially support one alternative in the sentence, but later on, another constraint strongly challenges that syntactic structure, the settling process is tremendously disrupted (Pickering and Van Gompel, 2006). Therefore, in *the constraint-based* account, there is a continuum of the *garden-path effect*; that is, it can disrupt comprehension to different degrees depending on the extent of competition among alternative interpretations leading to reading times ranging from fast to slow. However, based on the *syntax-first* models, a garden-path either happens (when the initial parse is incorrect) or it does not (when the parser selects the correct parsing option from the beginning).

### Ambiguity in prepositional phrases

Studies of sentence processing have made use of various forms of ambiguous structures to develop and assess models of syntactic processing. One of the most frequently investigated types of syntactic ambiguity involves the prepositional phrases (PPs) in V-NP-PP structures where there is more than one possible attachment site for the PP (i.e., NP and VP attachment). For example, in sentences like *The spy saw the cop with a revolver/binocular*, the reader will not be able to decide whether the PP is going to modify the NP or the VP until the noun in the PP unfolds. Then, relying on the world knowledge, the reader can assign the correct attachment to the PP. This temporary ambiguity is due to the incremental nature of language. There is consensus among all present models of sentence processing that language comprehension unfolds incrementally; that is, the language processing mechanism assigns an interpretation and a structure to every word as a constituent of a sentence as soon as they are encountered (Schütze, 1995; Ferreira and Cokal, 2015). There is a longstanding debate on how the human sentence processing mechanism selects one of the attachment types without knowledge of the incoming material.

According to the traditional *garden-path theory* (Frazier, 1979, 1987), the principles of *minimal attachment* and *late closure* can explain how the initial parsing decisions only rely on syntactic information. In a V-NP-PP sequence, the PP can have two functions: it modifies either the verb or the noun phrase preceding it. When a PP modifies the NP (known as *low or NP attachment*), an additional NP node must be constructed, which leads to a non-minimal attachment. Therefore, the preferred interpretation for the PP in this structure is the *VP attachment* also known as *high attachment*. However, when the PP is pragmatically inconsistent as a verb modifier, the syntactic processor is led down the garden path, and a reanalysis in favor of *NP attachment* is required resulting in longer reading times. The *garden-path theory* was corroborated by several studies (e.g., Ferreira and Clifton, 1986; Clifton and Ferreira, 1989; Britt et al., 1992; Rayner et al., 1992), but soon this kind of generalization to the human syntactic processing system was challenged by the proponents of the *constraint-based* approach. They argued that the syntactic bias suggested by *the garden-path theory* could not account for their findings, but that it is the content of the sentence that determines the attachment site (e.g., Taraban and McClelland, 1988; Trueswell et al., 1993). For instance, it has been shown that when there is a temporary ambiguity in a phrase between an argument and an adjunct, it takes adjuncts longer to be processed (Clifton et al., 1991). That is, in sentences like *The saleswoman tried to interest the man in the wallet/in his fifties during the storewide sale*, the prepositional phrase *in the wallet* which is an argument for the verb *interest* is processed faster than *in his fifties* which is an adjunct modifying *the man*. Furthermore, according to Abney (1989), the *minimal attachment* principle explains the attachment preference for arguments rather than adjuncts. Therefore, when studying attachment preferences, it is necessary to take into account whether the relationship between the PP and its potential attachment sites is of an adjunct or an argument type.

Another influential debate about the sources of information affecting parsing preferences concerns contextual cues. Crain and Steedman (1985) claimed that many parsing preferences occur due to the fact that sentences are presented out of context. They proposed the “referential theory,” according to which the parser builds the syntactic analysis of a constituent based on the semantic/pragmatic information available. Altmann and Steedman (1988) supported this theory and argued that in a VP-NP-PP sequence, the preferred attachment for the PP is explained by the presuppositions about the antecedents established in a prior context. In that study, they found that when a sentence such as *The burglar blew open the safe with the dynamite/new lock* is presented in isolation, both prepositional phrases *with the dynamite* and *with the new lock* would be initially parsed as a VP modifier, causing a *garden-path effect*. However, this preference changes when the readers are provided with a discourse context specifying whether the NP has one referent or more. In other words, when there is a unique referent for the NP, the PP is assumed to have a verb-modifying function, whereas when there is more than one referent, there is a tendency to clarify which referent is addressed, and hence, the PP will play a noun modifying role. Therefore, the preference for the verb-modifying function of the PP arises because, in the absence of prior context, only one referent is assumed for the NP.

Several other studies investigated the effect of referential context on parsing decisions (e.g., Trueswell and Tanenhaus, 1991; Altmann et al., 1992; Rayner et al., 1992; Britt, 1994; Murray and Liversedge, 1994; Altmann et al., 1998; Van Berkum et al., 1999), but the findings were contradictory. For instance, some of these studies found that prior context cannot avoid the *garden-path effect* in strongly biased structures. For instance, Britt (1994) manipulated two factors of referential ambiguity and verb argument structure to examine whether semantic processes also have a role in the initial parsing of a structurally ambiguous sentence. She used two groups of verbs differing in whether they take a PP as an optional or obligatory argument (e.g., *drop* vs. *put*) and found that the referential context neutralized the initial parsing preference for *high attachment* only in structures including verbs that required an optional PP argument (e.g., *He dropped the book on the chair before leaving*). However, when the structures contained a verb requiring an obligatory argument (e.g., *He put the book on the chair before leaving*), there was a more substantial syntactic bias for a *high attachment* which could not be overridden by the contextual cues.

### The interplay between contextual cues and syntactic parsing

Contextual information contributes to sentence comprehension mainly through three sources: intra-sentential, extra-sentential, and visual-situational (see Spivey et al., 2013 for a discussion). The third source of contextual cues, which is the focus of the present paper, refers to the non-linguistic information perceived visually in the immediate environment. There is some evidence that a visual context can affect the earliest stages of syntactic processing while listening to ambiguous structures. Tanenhaus et al. (1995) used an eye-movement monitoring study with the visual world paradigm to examine ambiguous double prepositional phrases such as *Put the apple on the towel in the box* and manipulated the contextual cues by providing a two-referent (containing two apples) and a one-referent context (containing one apple). They observed that the existence of two referents in the context could guide the parser toward an NP attachment which is assumed to be the less preferred reading. However, Snedeker and Trueswell’s (2004) findings in a similar experiment containing *with-PP* structures (e.g., *Tickle the pig with the fan*) contradict those of Tanenhaus et al. (1995). They attributed this to the distinctive semantic properties of the PPs used in the two studies (i.e., instrumental vs. locative PPs). Furthermore, Chambers et al. (2004) reported that certain higher-level pragmatic constraints, such as communicative intentions or expectations based on the world knowledge, can also influence the syntactic parsing preferences.

It is conceivable that during sentence interpretation, readers/listeners generate forms of mental images of events in propositions. A number of studies have suggested that merely imagining or recalling the information associated with absent stimuli can affect eye movements while processing a sentence (see Huettig et al., 2011 for a review). Altmann (2004) found that a recent memory of the visual stimuli can lead to the formulation of an interpretation during online sentence processing. Altmann and Kamide (2009) investigated the role of prediction during language processing and found that the mental representation that is shaped while processing linguistic input and a visual scene is a better representative of the listeners’ anticipatory and concurrent eye movements than the properties of spoken input and the visual scene independent from each other. Furthermore, Christie et al. (2017) used images as a means of resolving prepositional phrase attachment ambiguity and found that visually aided contextual information strongly guides sentence interpretation during sentence processing. Berzak et al. (2016) used videos as a visual context to resolve semantic-, syntactic-, or discourse-level ambiguities, including prepositional phrase attachment. They found that visual aid helps participants resolve ambiguities.

Taken together, the above-mentioned studies (i.e., Tanenhaus et al., 1995; Altmann, 2004; Snedeker and Trueswell, 2004; Altmann and Kamide, 2009) have shown that visually enhanced contextual information can guide the parser during structural analysis toward a particular interpretation, even if the scene is removed before presenting the structure. However, studies looking at both visual contextual information and verb argument structure are scant. Furthermore, studies investigating initial parsing preferences have mostly used the visual world paradigm with spoken stimulus, leaving the reading modality somewhat unexplored in the presence of visual contexts. This is the topic of the current study. We utilized a visually enhanced self-paced reading task, in which contextual information is visually depicted by means of short animation clips. This paradigm allowed us to record readers’ word-by-word reading times after they were guided with visual information toward a certain structural analysis.

### The present study

This study focuses on ambiguity resolution in Dutch double PP constructions with two possible attachment sites for the PPs; see (1) for an example.

a. *Low/NP attachment*

Ze zet _NP_[_NP_[de klok] _PP1_[naast de foto]] _PP2_[op de tafel].

She puts _NP_[_NP_[the clock] _PP1_[next to the photo]] _PP2_[on the table].

“She puts the clock that is next to the photo on the table.”

b. *High/VP attachment*

Ze zet _NP_[de klok] _PP_[_PP1_[naast de foto _PP2_[op de tafel]].

She puts _NP_[the clock] _PP_[_PP1_[next to the photo _PP2_[on the table]].

“She puts the clock next to the photo that is on the table.”

In order to clearly distinguish between the two potential interpretations, we apply the terms *high* and *low* attachment to these interpretations. Such use of *high/low attachment* is often encountered in constructions such as VP NP PP which contain only one ambiguously attached PP. If the PP is attached to the VP, it is called *high attachment*, whereas *low attachment* implies the PP is attached to the NP. We extended the use of these terms to double PP constructions by taking the attachment of the first PP to determine whether the structure is of a *high* or *low* attachment type. More precisely, if the first PP attaches to the noun (1), we call it *low attachment*, and if it attaches to the verb (2), we call it *high attachment*. Despite the general tendency in the literature to use the terms *high/low attachment* for VP NP PP structures, our studies focus exclusively on the processing of double PP structures.

Given that these structures are globally ambiguous, disambiguation cannot happen by solely relying on the linguistic information within the sentence. As discussed earlier, there is strong evidence confirming the efficacy of a visual context as a means of ambiguity resolution either when presented simultaneously with the linguistic stimuli or before it. In this kind of context, disambiguation occurs by drawing on memory and the mental representations shaped while the visual context is presented. Therefore, in the current study, we presented animation clips before presenting a written sentence to guide syntactic parsing. According to the previous research (e.g., Tanenhaus et al., 1995), the parser seems to have a general preference for *high attachment* while processing a PP attachment ambiguity in structures with locative PPs. The proponents of *syntax-first* models attribute this preference to the *minimal attachment* principle (Frazier, 1979) and the *constraint-based* account explains that this preference is because, in a competition among different constraints (e.g., frequency of use and verb bias), *high attachment* interpretation receives the highest amount of support and is, therefore, the preferred interpretation.^1^

As discussed earlier, what distinguishes *syntax-first* and *constraint-based* models is their assumptions about the time course of activation of different information sources. That is, according to the *syntax-first* model, only syntactic information is initially activated, whereas in the *constraint-based* model, different sources of information interact from the earliest stages of analysis. One aim of this study is to investigate whether non-syntactic information (i.e., contextual cues) can affect the initial analysis of a sentence. Particularly, we aim to examine whether we can avoid being garden-pathed by presenting a visual context prior to reading an ambiguous structure. If the contextual cues can override the general syntactic preference for *high attachment* in this type of constructions, we expect to observe no difference in reading times between the *high* and *low attachment* interpretations. However, if syntactic preferences are stronger than the contextual cues, it is expected that when the clips guide the readers toward a *low attachment* interpretation, there is a slowdown in reading at PP2. This disruption is due to the initial assignment of a *high attachment* interpretation to PP1, and a need for reanalysis when facing PP2. It is assumed that reanalysis happens as a result of a mismatch between the interpretation raised by contextual cues (*low attachment*) and the generally preferred *high attachment* interpretation analyses (Frazier and Rayner, 1982; Ferreira and Clifton, 1986; Tanenhaus et al., 1995).

Despite the critical role of the subcategorization information of the verb during parsing, only a few studies have compared verbs with different argument structures in a single study (e.g., Britt, 1994). This motivated the second aim of the present study: to examine whether the verb’s argument structure influences the extent to which a visual context can guide parsing. In this study, we used two types of verbs differing in their argument structures: 3-Argument Verbs and 2-Argument Verbs (see the *design and materials* section for more details on verb types). Given that verb bias has been reported to influence the interpretation of ambiguous structures (e.g., Trueswell et al., 1993; Britt, 1994; MacDonald, 1994; Garnsey et al., 1997; Staub, 2007), we expect that 3-Argument Verbs will have a stronger preference for *high attachment*; therefore, the disambiguation toward a *low attachment* interpretation is expected to impose a higher processing load on the parser, leading to longer reading times in the critical region (i.e., PP2). Furthermore, 3-Argument Verbs were of two types based on the obligatory or optional nature of the PP attachment as an argument. According to Britt (1994), we expect to observe a stronger preference for *high attachment* reading of structures including obligatory PPs, leading to longer reading times while processing *low attachment* interpretations including obligatory PPs.

## Methodology

### Participants

Forty-six right-handed native speakers of Dutch (25 female, 21 male, mean age = 22.69, range = 18–30, SD = 2.99) with no history of neurological/psychiatric issues or hearing impairment participated in this experiment. The participants were residents in the city of Groningen at the time of their participation. They signed informed consent prior to participating in this study confirming that their participation was voluntary and received 8 euros for their participation. This study has been approved by the Research Ethics Committee (CETO) of the Faculty of Arts, University of Groningen (CETO approval date and number: 02/09/2019–65,065,359).

### Design and materials

The experimental materials consisted of 80 Dutch globally ambiguous double PP structures, with two possible attachment sites for the PPs: *VP* and *NP attachment* also known as *high* and *low attachment* (we use the latter terms in the remainder of the paper). The items were divided into two groups based on the argument structure of the verbs: the 2-Argument Verbs required a subject NP and an object NP (e.g., *openen:* “to open”*),* while the 3-Argument Verbs required a locative PP in addition to the NPs (e.g., *zetten:* “to put” and *verstoppen*: “to hide”). The verbs in this group varied regarding their need for an optional or an obligatory PP. For instance, a sentence with the verb *zetten* (*to put*) is ungrammatical if it lacks a locative PP, while a verb such as *verstoppen* (*to hide*) implicitly requires a PP, but a sentence is still grammatical in its absence. To avoid sentence-final wrap-up effects and to be able to measure possible spillover effects, we added a conjunction sentence to the end of the items (e.g., *Hij opent het boek naast de lamp op het bureau en gaat het lezen*. “He opens the book next to the lamp on the desk and starts reading it.”). Experimental sentences were distributed over two unique sets of sentences so that each participant encountered a sentence either in *high* or *low attachment* reading. Each list was composed of 80 fillers and 80 experimental items distributed pseudorandomly across four blocks, within which there were five experimental items per condition and 20 fillers.

The experiment consisted of two factors each including two levels. That is, the factor of Attachment Type, which the accompanying clip biased toward, consisted of two levels of High and Low, and the factor of Argument Structure included two levels of 2-Argument and 3-Argument Verbs. Therefore, as presented in Table 1, these factors formed four conditions in the experiment as follows: High Attachment/3-Argument Verbs, Low Attachment/3-Argument Verbs, High Attachment/2-Argument Verbs, and Low Attachment/2-Argument Verbs. The dependent variable was the reading time per region of interest including R1: the noun preceding PP2, R2: The preposition in PP2, R3: the article in PP2, R4: the noun in PP2, R5: the spillover region, and R6: the final word (see Table 2). It is noteworthy that the experimental sentences had the same structure and length up to the spillover region which always started with a conjunction and was the same in both the *high* and *low attachment* versions of each sentence.

**Table 1:** The conditions for the presentation of experimental items.

Condition	Example				
HA\3AV	Ze	zet	_NP_[de klok]	_PP_[_PP1_[naast de foto]	_PP2_[op de tafel]]
	she	puts	_NP_[the clock]	_PP_[_PP1_[next to the photo]	_PP2_[on the table]]
	“She puts the clock next to the photo that is on the table.”				
LA\3AV	Ze	zet	_NP_[_NP_[de klok]	_PP1_[naast de foto]]	_PP2_[op de tafel]
	she	puts	_NP_[_NP_[the clock]	_PP1_[next to the photo]]	_PP2_[on the table]
	“She puts the clock that is next to the photo on the table.”				
HA\2AV	Hij	opent	_NP_[het boek]	_PP_[_PP1_[naast de lamp]	_PP2_[op het bureau]]
	He	opens	_NP_[the book]	_PP_[_PP1_[next to the lamp]	_PP2_[on the desk]]
	“He opens the book next to the lamp that is on the desk.”				
LA\2AV	Hij	opent	_NP_[_NP_[het boek]	_PP1_[naast de lamp]]	_PP2_[op het bureau]
	He	opens	_NP_[_NP_[the book]	_PP1_[next to the lamp]]	_PP2_[on the desk]
	“He opens the book that is next to the lamp on the desk.”				

The conditions are abbreviated as HA/3AV, High Attachment/3-Argument Verbs; LA/3AV, Low Attachment/3-Argument Verbs; HA/2AV, High Attachment/2-Argument Verbs; LA/2AV, Low Attachment/2-Argument Verbs.

**Table 2:** The regions of interest in the structures under study.

*Region*	*Precritical regions*						*R1*	*R2*	*R3*	*R4*	*R5*	*R6*
*Example*	Ze/	zet/	de/	klok/	naast/	de/	foto/	op/	de/	tafel/	[SO]/	[FW]/
*Eng.*	She/	puts/	the/	clock/	next to/	the/	photo/	on/	the/	table/	[SO]/	[FW]/

R1, The noun preceding the second PP; R2, The preposition in the second PP; R3, The article in the second PP; R4, The noun in the second PP; R5, The spillover region (SO); R6, The final word (FW); The sign “/” separates individual regions.

As the ambiguous sentences could have both *high* and *low attachment* readings, we used animations to provide bias for a desired interpretation in a given trial. To create these guiding animations, we used an online program^2^ to develop 160 animation clips for the experimental items (i.e., one animation for the *high attachment* interpretation and one animation for the *low attachment* interpretation per experimental trial) and 80 clips representing the filler items. All the clips were silent and lasted from 6 to 9 s. We designed each pair of clips to represent one ambiguous sentence using a similar setting, character, and direction of movement. It is noteworthy that the experimental design did not include conditions in which the animation clips were absent because, given the global nature of ambiguity in the structures under study, it was not possible to draw conclusions from the reading times of these structures.

**Figure 1:**
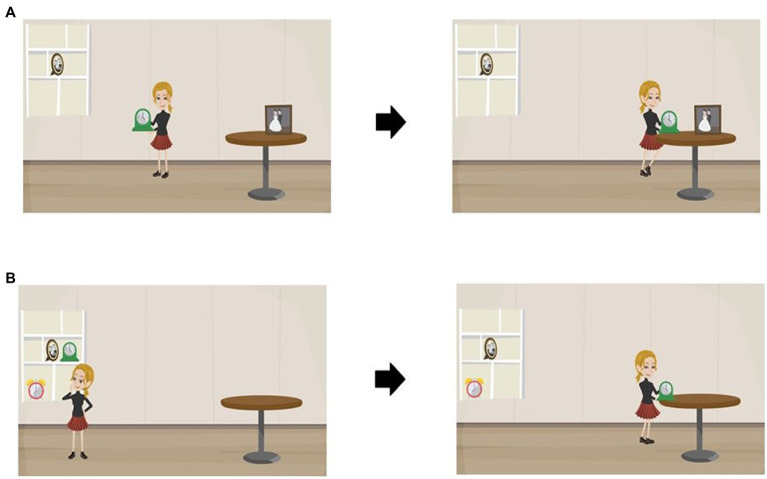
Parts of the animations representing two different attachment types for the sentence “Ze zet de klok naast de foto op de tafel” (She puts the clock next to the photo on the table): **(A)** High-Attachment interpretation. **(B)** Low-Attachment interpretation.

As shown in Figure 1, each experimental clip depicted a character who sees two similar objects placed in different locations. These objects represent the noun following the verb and the noun in the first PP. For instance, in the sentence *Ze zet de klok naast de foto op de tafel: “*She puts the clock next to the photo on the table”, in the clip representing the *high attachment* reading (A), there is one clock and two photos, and the girl chooses to put the clock next to the photo that is on the table. However, in the clip that shows the *low attachment* interpretation (B), there are two clocks, one of which is next to the photo, and the character places the clock that is next to the photo on the table. The idea behind having the character decide between two clocks or two photos in each clip comes from Crain and Steedman’s (1985) “referential theory.” According to this theory, when there is more than one referent for a noun, there is a tendency to specify which referent is meant, therefore there will be a need for a PP to modify the noun. However, if there is a unique referent for the NP, there is no need for the PP to modify the noun, and it will modify the verb instead. Hence, in the example mentioned above, the PPs once describe the clock and once the photo.

### Procedure

The self-paced reading task and animation clips were presented in EPrime 2.0 (Psychology Software Tools, Inc.), using a word-by-word moving window paradigm. In this paradigm, the whole sentence is outlined on the screen with a series of dashes, marking the position and length of the words in the sentence. Words appeared in linear succession on the screen: When participants pressed the spacebar, dashes were replaced with words one at a time, with each word reverting back to a dash when the button was pressed for the next word. Each trial began with a one-second fixation cross. Participants were instructed to read at a normal speed. The final word was always marked with a full stop. The amount of non-cumulative time spent reading each segment was recorded as the time between key presses. To ensure that participants read the sentences for meaning and also paid attention to the animation clips, we presented yes-no questions pseudorandomly across the experiment after one-quarter of all of the sentences (i.e., both experimental and filler items). Participants were required to press the appropriate key on a keyboard (“p” or “q”) depending on whether the answer to the question was “yes” or “no,” followed by feedback indicating “correct” or “incorrect” appearing in green and red, respectively, on a black screen for 500 ms. Half of the questions were correctly answered with a “yes” response, and half with a “no” response. In order to keep the attention of the participants to both the animations and the written stimuli, we designed three types of questions: questions that targeted the information that was merely found in the written stimuli (e.g., questions about what the character thought in a given situation); questions that only needed attention to the animations (e.g., the color of the character’s hair); and questions that could be answered both through the animations and the written sentences. This was explained to the participants prior to the experiment, and they practiced the questions during the practice block. Additionally, they were also informed that the aim of the study was to investigate the effect of the animation clips on how they read the sentences. Such information was provided so that participants would try to keep the animations in their minds while reading the sentences. We assumed that having such information regarding the aim of the study would avoid confusion and enhance concentration for participants. There was one practice block of eight items presented before the experimental blocks in order to familiarize the participants with the task. The participants could take a break for as long as they needed after completing each of the four blocks. The whole experiment lasted around 1 h.

### Data analysis

In the first step, the accuracy of the participants’ performance on the comprehension questions was examined, and only those participants who scored at least 80% correct on these questions were included in the analysis (Witzel et al., 2012). Only one participant who did not meet the criteria (accuracy: 72%) was excluded. Next, we applied *a priori* cut-offs for reading/reaction times, excluding durations below 100 ms and above 4,000 ms. These cut-offs removed 0.14% of the remaining data. Further outlier exclusion was performed based on the statistical models presented in the following section.

The data were analyzed in R (version 4.1.1; R Core Team, 2021). To investigate the possible effects of our factors of interest on processing speed, linear mixed-effects regression models were fitted with reaction time (RT) as the dependent variable using the *lmer* function of the *lme4* package (version 1.1–27.1; Bates et al., 2015). The models were constructed with the hypothesis testing fixed effect of Attachment Type (*high* vs. *low*) on reading time of structures including *2-Argument Verbs* and *3-Argument Verbs*. The binary predictor of Attachment Type was sum-to-zero coded (i.e., +0.5 or −0.5) to avoid biases due to data imbalance. The control predictor of Word Length was mean-centered to avoid multicollinearity issues which could affect model convergence and/or inflate the standard errors. Moreover, we performed model comparison to obtain optimal model structures based on model fit using Analysis of Variance (ANOVA).

Reading times were log-transformed after *a priori* cut-off application of ≥ 100 and ≤ 4,000 ms to conform to the normality of residuals distribution in linear mixed-effects models. The log (RT) models incorporated the hypothesis testing effect described earlier. The Word Length was introduced to the models to control for this objective measure. Participants’ age and sex were used as exploratory fixed effects but did not improve the model. Experimental participants and items/trials were added as random intercepts and slopes where appropriate and contributed to the model (Baayen, 2008). Statistical significance was set to *p* < 0.05, and the *p*-values were calculated based on the Satterthwaite approximation using the *lmerTest* package (Kuznetsova et al., 2017). Furthermore, as stated earlier, the *3-Argument Verbs* in our study were twofold in terms of whether they required an obligatory or optional argument. Therefore, we included the *Verb Type* as an exploratory fixed effect. However, exploration of verb type did not improve the model fit significantly as measured through model comparison and therefore was excluded from the final model. The regions of interest were selected drawing upon the previous studies (e.g., Britt, 1994; Tanenhaus et al., 1995; Spivey et al., 2002; Chambers et al., 2004; Boudewyn et al., 2014) whereby the second PP was considered as the point the reanalysis occurred. Additionally, we included the word preceding PP2 as the precritical region and the spillover region and the final word as the postcritical region. With the inclusion of these regions, we aimed to examine from/to which region in the structure the visual context would influence processing, in case there was an effect. Even though the predetermined regions of our interest included the noun preceding PP2, PP2, the spillover region, and the final word, we initially ran a global analysis with the inclusion of *Attachment Type* and *Argument Structure* for every word in the sentence. This step was taken to examine whether the visual context affected the processing of these structures in the regions preceding our regions of interest (i.e., R1 to R6, see Table 2). This extra step was taken to make sure the visual context does not affect the processing earlier in the sentence as, to our knowledge, no previous study has used similar manipulations in a reading study. Then, we subset the data based on the argument structure and analyzed the data separately per critical region, once for 2-Argument Structures and once for 3-Argument structures with the inclusion of *Attachment Type* as a fixed effect.

## Results

**Figure 2:**
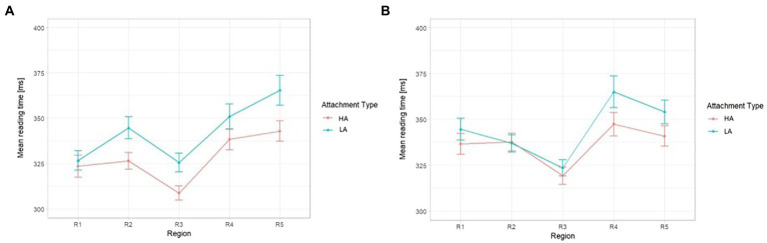
Trimmed mean reading times [ms] for the High- and Low-Attachment conditions across the critical regions: **(A)** 3-argument verbs, **(B)** 2-argument verbs.

**Figure 3:**
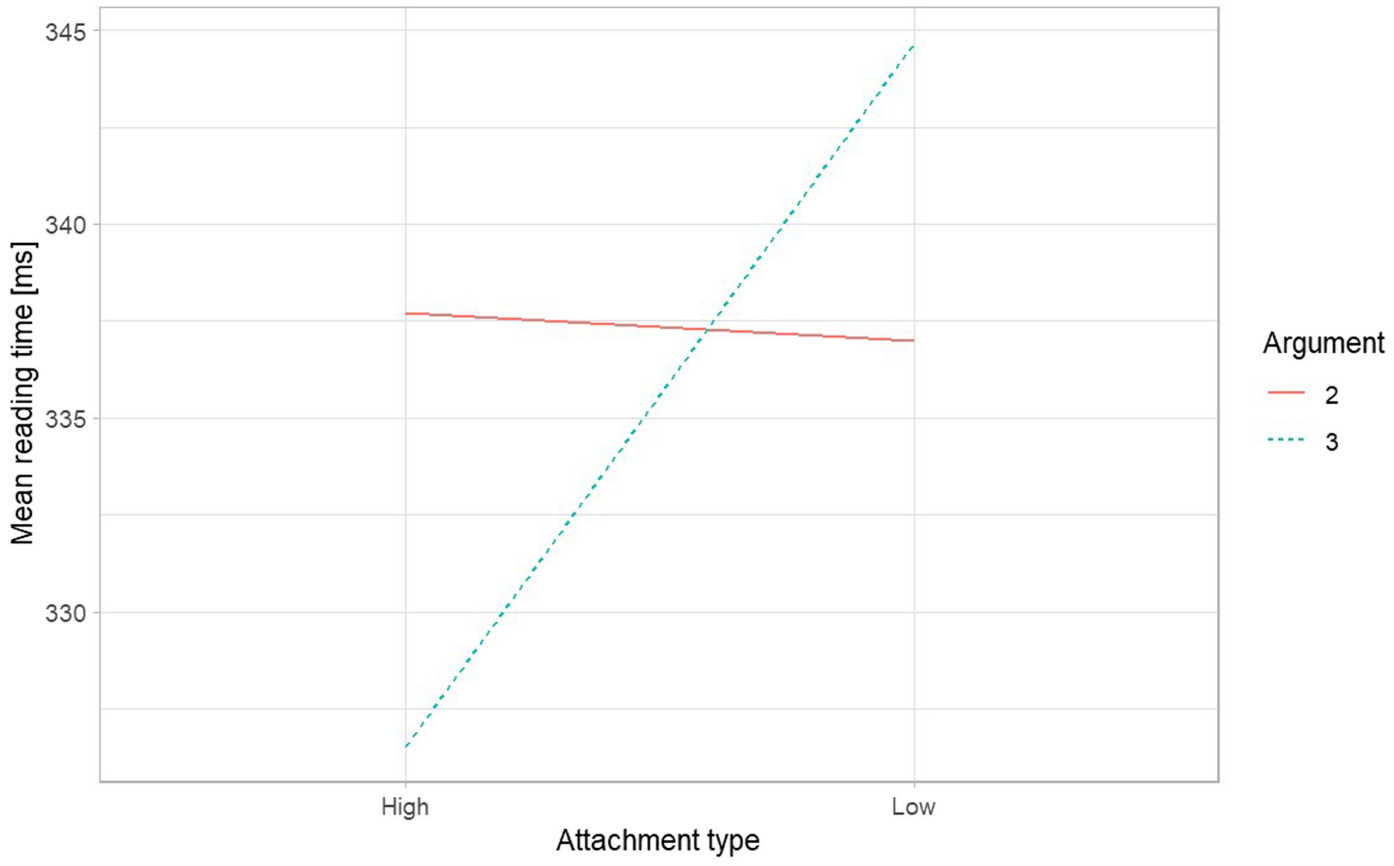
The interaction plot from the global model for region 2: the preposition in PP2.

Mean reading times per region of interest across the four conditions are given in Table 3; Figure 2. The global model showed that there were no significant effects of *Attachment Type* and *Argument Structure* nor their interaction across the sentence up to R2 (the preposition in PP2) where, as displayed in Figure 3, the model yielded a significant interaction between the two fixed effects of *Attachment Type* and *Argument Structure* (*ß* = 0.03, SE = 0.01, *t* = 2.19, *p* = 0.028), while there were no significant fixed effects of *Attachment Type* (*ß* = 0.01, SE = 0.006, *t* = 1.50, *p* = 0.13), nor of *Argument Structure* (*ß* = −0.02, SE = 0.02, *t* = −0.83, *p* = 0.41) in that region. In the following regions, the global models only pointed to significant effects of *Attachment Type* (R3: *ß* = 0.02, SE = 0.006, *t* = 4.25, *p* < 0.001; R4: *ß* = 0.02, SE = 0.01, *t* = 2.99, *p* = 0.002; R5: *ß* = 0.015, SE = 0.006, *t* = 2.22, *p* = 0.026). All other fixed effects including condition manipulations returned non-significant (see Supplementary Appendix 1). Given these outputs, across the sentence from R3 (the article in PP2) onward, the participants experienced a reading disruption in Low Attachment conditions without a significant modulation of verb argument structure. We, therefore, subset the data at this point and carried on analyzing per verb argument structure separately.

**Table 3:** The mean and SD of the trimmed raw reading times [ms] per region per condition.

Region	HA/3AV		LA/3AV		HA/2AV		LA/2AV	
	Mean	SD	Mean	SD	Mean	SD	Mean	SD
R1	323.59	183.1	326.66	160.36	336.60	170.16	344.68	179.29
R2	326.52	138.52	344.67	182.24	337.72	145.84	336.97	141.23
R3	308.78	117.51	325.55	154.79	319.31	139.37	323.60	130.96
R4	338.39	175.1	351.01	210.14	347.40	192.76	365.08	260.03
R5	342.94	167.61	365.46	246.25	340.96	169.81	354.10	194.41
R6	487.21	284.54	519.37	368.84	497.35	294.64	506.17	334.74

### 3-argument verbs

In R1 (i.e., the noun preceding PP2), there was no significant effect of *Attachment Type* (*ß* = 0.006, SE = 0.012, *t* = 0.534, *p* = 0.594) or *Word Length* (*ß* = −0.007, SE = 0.009, *t* = −0.734, *p* = 0.467). However, in R2 that comprised the preposition in the second prepositional phrase, the Low-Attachment/3-Argument condition was read more slowly than the High-Attachment/3-Argument condition (*ß* = 0.033, SE = 0.012, *t* = 2.707, *p* = 0.006) as was also the case in R3 that comprised the article in the second prepositional phrase (*ß* = 0.040, SE = 0.011, *t* = 3.626, *p* < 0.001) In R4 (i.e., the noun in the second prepositional phrase) only a marginal effect of attachment type was observed (*ß* = 0.025, SE = 0.013, *t* = 1.865, *p* = 0.062). Nevertheless, in R5 being the spillover region (*ß* = 0.029, SE = 0.014, *t* = 2.050, *p* = 0.04) and R6 representing the final word (*ß* = 0.041, SE = 0.016, *t* = 2.495, *p* = 0.012), we, once again, observed significantly slower reading times for the *Low-Attachment/3-Argument* condition. Concerning the Word Length variable, no significant effect was observed across the whole region of interest including R1 (*ß* = −0.007, SE = 0.009, *t* = −0.734, *p* = 0.467), R2 (*ß* = −0.018, SE = 0.009, *t* = −1.916, *p* = 0.061), R3 (*ß* = 0.027, SE = 0.032, *t* = 0.852, *p* = 0.399), R4 (*ß* = 0.014, SE = 0.011, *t* = 1.236, *p* = 0.224), R5 (*ß* = −0.017, SE = 0.024, *t* = −0.715, *p* = 0.478), and R6 (*ß* = 0.019, SE = 0.013, *t* = 1.444, *p* = 0.156).

### 2-argument verbs

The statistical analysis revealed no significant effect of *Attachment Type* in R1 (*ß* = 0.014, SE = 0.012, *t* = 1.126, *p* = 0.2603), R2 (*ß* = −0.003, SE = 0.012, *t* = −0.273, *p* = 0.785), R3 (*ß* = 0.017, SE = 0.010, *t* = 1.634, *p* = 0.103), R4 (*ß* = 0.020, SE = 0.014, *t* = 1.398, *p* = 0.1623), and R6 (*ß* = 0.012, SE = 0.016, *t* = 0.752, *p* = 0.452). However, at R5 representing the spillover region there was a marginal effect of *Attachment Type* showing a preference for High-Attachment (*ß* = 0.023, SE = 0.012, *t* = 1.836, *p* = 0.066). Furthermore, the analysis yielded a significant effect for *Word Length* in R1 being the noun preceding PP2 (*ß* = 0.022, SE = 0.011, *t* = 2.027, *p* = 0.049) and R4 representing the noun in PP2 (*ß* = 0.030, SE = 0.013, *t* = 2.241, *p* = 0.03). For all other regions of interest, the fixed effect of *Word Length* returned non-significant R2 (*ß* = −0.017, SE = 0.015, *t* = −1.154, *p* = 0.255), R3 (*ß* = 0.023, SE = 0.039, *t* = 0.597, *p* = 0.554), R5 (*ß* = −0.021, SE = 0.032, *t* = −0.677, *p* = 0.502), R6 (*ß* = −0.013, SE = 0.013, *t* = −0.983, *p* = 0.33).

Summarizing, our data revealed different processing behaviors while reading 2-Argument and 3-Argument structures. Lack of a significant difference in reading times of conditions High-Attachment/2-Argument and Low-Attachment/2-Argument can be taken as evidence that the animation clips presented before the reading task have been able to prevent the *garden-path effect.* That is, the contextual cues seem to have successfully overcome the preference for the *high attachment* interpretation. This preference is generally observed by a higher processing load leading to longer reading times in the *low attachment* reading. However, this was not the case with the 3-Argument conditions where the *low attachment* interpretation led to reading disruptions with longer reading times in PP2, the spillover region, and the final word. As we were not initially interested in the interaction between two factors of Attachment Type and Argument Structure, and the global model yielded an interaction effect only in one region in the sentence, in the following section, we will only focus on the main effect of Attachment Type observed in the latter analysis.

## Discussion

The aim of this study was to investigate (i) whether we can prevent a reader from being garden-pathed by presenting an animated visual context prior to a reading task and (ii) whether the verb’s argument structure influences readers’ preference for one attachment type in the presence of a disambiguating visual context. Our findings revealed that the visual context presented before the reading tasks does guide the initial parsing depending on the argument structure of the verb. That is, in structures including 2-Argument verbs, both *high* and *low attachment* interpretations were read at a similar pace; however, when the verb showed a strong bias for a PP, as with structures including 3-Argument verbs, the readers showed a strong preference for *high attachment*. We suspect that the facilitated reading of 2-Argument structures can be due to the efficiency of the contextual cues in guiding the syntactic analysis toward the desired interpretation. Below, we will discuss these findings in the light of the current theories of sentence processing.

Regarding the first aim, it is important to establish whether visual contextual information can override the attachment preference caused by syntactic information during sentence processing. Following the modularity assumption of the *syntax-first* model (Frazier, 1979, 1987; Frazier and Rayner, 1982) which states that the initial parsing decisions are made merely by relying on syntactic information, we expect that the contextual cues do not affect the choice of attachment site for the PP. Alternatively, drawing on the *constraint-based* account (Trueswell et al., 1993; McRae et al., 1998) where it is assumed that different interpretations are activated in parallel and are in competition with each other, we would expect the contextual cues to influence parsing.

Under the *syntax-first* account and based on the *minimal attachment* principle, in the structures under study (i.e., V-NP-PP sequence), High-Attachment would be the preferred syntactic analysis, irrespective of the visual context preceding it and the lexical properties of the verb. The results from the present study suggest that despite the presence of a disambiguating context, the readers were still garden-pathed. However, a closer look revealed that this occurred only when they read 3-Argument structures. That is, 3-Argument/Low-Attachment condition was processed more slowly than 3-Argument/High-Attachment condition whereas there was no significant difference in reading times of 2-Argument/Low-Attachment and 2-Argument/High-Attachment conditions. Serial models such as *syntax-first* regard such slowdown in the reading rate of 3-Argument structures as a sign of reanalysis. Despite the presence of contextual cues aiming to guide parsing toward the intended interpretation, the participants in this study still initially attached the PP to the VP (i.e., High-Attachment). At a later stage, drawing upon the mental representations formed by watching the animation clip showing a Low-Attachment interpretation, they realized a mismatch between their own High-Attachment reading and the interpretation formed by the contextual cues leading to a reanalysis. According to our data, the reanalysis occurred at the second PP and continued all the way to the spillover region and the final word in structures including 3-Argument Verbs. Therefore, in the case of 3-Argument structures, syntactic relations supporting *minimal attachment* take precedence over contextual information. However, the predictions of the traditional Garden-path theory cannot account for the processing of 2-Argument structures where a similar processing behavior for High-Attachment and Low-Attachment interpretations was observed. Indeed, lack of a preference for one attachment site would mean either that the contextual cues interact with the syntactic information and guide parsing which is against the assumptions of serial processing, or that attachment is underspecified in these structures. The latter possibility has been accounted for in *construal theory*.

*Construal theory* (Frazier and Clifton, 1996, 1997) which is the refined version of the garden-path model, seems to better account for the different processing patterns of 3-Argument and 2-Argument structures under investigation. According to *construal theory*, syntactic structures are divided into primary (i.e., the complements and obligatory constituents of a phrase) and non-primary phrases (i.e., optional constituents of a phrase), and the principles of *late closure* and *minimal attachment* only apply to primary relations. In other words, non-primary relations are assigned an underspecified analysis because they are only *associated* with a thematic processing domain rather than being attached to it. In the current study, because of the verb’s bias for a complement locative PP, 3-Argument structures are expected to instigate primary relations when the verb is known, leading to the application of *minimal attachment* principle and hence a preference for a High-Attachment interpretation. In 2-Argument structures, however, the argument structure of the verb does not require a PP, and thus forms a non-primary phrase with an underspecified analysis (i.e., no preference for either attachment site). It is likely that the similar processing behavior of participants while reading High-Attachment/2-Argument and Low-Attachment/2-Argument conditions is evidence for the underspecification of nonprimary relations. However, one shortcoming of *construal theory* is that it cannot identify the exact time course for the processing of nonprimary phrases (e.g., at what stage semantic and pragmatic cues affect parsing; Papadopoulou, 2006). This limitation is also evident in the present study; we cannot determine whether lack of a preference for an attachment site in 2-Argument structures is simply due to indeterministic parsing of these structures, or whether the contextual cues are directing parsing from the earliest stages of processing.

Serial models discussed in this paper (Frazier, 1979, 1987; Frazier and Clifton, 1996, 1997) can only partly account for our findings. That is to say, it can be claimed that in the processing of 3-Argument structures, there is initially a default syntactic preference for High-Attachment, but at a later stage, when participants realize there is a mismatch between the mental representations formed by the animation clip and the preferred High-Attachment interpretation, reanalysis occurs. Nevertheless, lack of an attachment preference in 2-Argument structures suggests that there is an interaction between the contextual cues and syntactic information from the earliest stages of processing. Parallel activation of different sources of information can be discussed in the light of *constraint-based* model (MacDonald, 1994; Tanenhaus et al., 1995), which seems capable of adequately addressing the first two aims of this study. Based on this model, the animation clips in the present study provide a contextual constraint that is in competition with other constraints such as syntactic bias and verb subcategorization information. Depending on the extent to which these constraints guide the intended interpretation, reading can be facilitated or disrupted. For instance, in 3-Argument structures where there is a general syntactic bias for High-Attachment, if the animation clip supports a High-Attachment interpretation, processing would be faster than when it provides a Low-Attachment interpretation. Therefore, distinctive processing patterns observed in 2-Argument vs. 3-Argument structures can be attributed to differing levels of competition among the available constraints, namely attachment preference, verbs’ argument structure, and contextual cues that are actively graded during the initial stages of parsing. Determining the strength of these constraints, however, requires the implementation of computational modeling which is outside the scope of this study.

In *constraint-based* model (MacDonald, 1994; Tanenhaus et al., 1995), the probabilistic nature of constraints warrants the simultaneous activation of alternative structures that are in competition with each other. As discussed earlier, depending on the degree of support for each constraint, competition between different interpretations can take longer or shorter, substantiating the existence of a *garden-path effect* continuum, whereas in *syntax-first* approach, the *garden-path effect* either occurs or it does not. Our data showed that in 3-Argument structures reading was disrupted from R2 to R6 while in 2-Argument structures we found a marginal effect only in R5. Taking into account the marginal effect of attachment type in 2-Argument structures, it can be cautiously claimed that competition between different constraints was resolved faster in 2-Argument structures than in 3-Argument structures, supporting the existence of a *garden-path effect* continuum.

Another interactive approach toward discussing our findings regards the referential theory of Crain and Steedman (1985). Based on this theory, the general preference for High-Attachment is due to the presupposition for the existence of a single referent for a noun in a null context. In other words, when there is only one referent for a noun, there is no need to modify it (Low-Attachment) since it provides redundant information and violates the Gricean maxim of quantity: give as much information as needed, and no more (Grice, 1975). In the present study, we used animation clips to provide two referents once for the object NP (de klok: “*the clock*” in 1) to facilitate Low-Attachment interpretation and once for the noun in PP1 (de foto: “*the photo*” in 1) to guide the readers toward a High-Attachment interpretation. Given the explicit presentation of two referents in the animation clips, we expected that it would overcome the general preference for High-Attachment. However, our data showed that such a preference can be avoided only in 2-Argument structures suggesting that in a competition between syntactic and contextual constraints, syntactic bias is stronger than contextual bias. Britt (1994) also found that the lexical properties of verbs determine whether the referential context could neutralize the parsing preference for High-Attachment or not. Britt used two types of 3-Argument structures differing in the obligatory and optional nature of the PP as the verb’s argument (e.g., *put*, vs. *drop*). She found that contextual cues could only neutralize the parsing preference for High-Attachment when the PP was an optional argument. Both Britt’s and the present study provide support for the important role of the lexical properties of verb in making syntactic decisions during the initial analysis^3^.

Several studies have so far investigated the initial parsing preferences in structures with PP attachment ambiguity. Despite the fact that all of these studies focused on the same syntactic structure, the *garden-path effect* was observed only in some of them after manipulating the contextual cues. For instance, while similar to our findings, Britt (1994) and Snedeker and Trueswell (2004) found that the two-referent scenes could not completely eliminate the VP attachment preference, other studies (e.g., Tanenhaus et al., 1995) reported that a two-referent context could neutralize the attachment preference. Such diversity in findings can be attributed to the nuances in the design of different studies, including the lexico-grammatical properties of the verbs, the semantic properties of the PPs (e.g., instrument vs. location sense), the mode of presentation (e.g., auditory vs. written), the type of ambiguity (e.g., local vs. global), and the means of disambiguation [e.g., prior extra-sentential discourse, simultaneous visual cues (VWP), and prior visual context (animation clips)]. The sensitivity of the findings to the design gives evidence supporting the contribution of multiple sources of information to the computation of a syntactic assignment from the initial stages of analysis and challenges the theories supporting the autonomy of syntax and serial processing. In this study, the significant difference observed in the processing of High-Attachment and Low-Attachment structures gives evidence that the visual context presented prior to reading could successfully disambiguate the globally ambiguous structures and provided support for the facilitatory role of vision in the form of images and videos in language processing, in general, and ambiguity resolution, in particular as previously found by Berzak et al. (2016) and Christie et al. (2017). This finding also supports Altmann’s (2004) study, showing that recent memory of visual stimuli affects the formulation of an interpretation.

We would also like to address the limitations of the current study. One issue that we glossed over is that in the case of two-argument verbs, there is an additional reading in the form of V NP, in which the NP contains both PPs. This option was not included in the original design, since the study focuses on the relationship between the verb and its arguments. The attachment inside the NP is not related to the verb argument structure. However, in the unlikely case that this parsing option is the default option, we would expect to see reanalysis effects in both high and low attachment readings in our paradigm. The effect should be registered at PP1 for high attachment and PP2 for low attachment. Since there were no differences between *high* and *low* attachment reading for 2-argument verbs, we assume that the V NP option is not the default option. At this point, we assume that the presence of said option did not play any role in the processing investigated in the current study. However, it is an issue that may add an additional layer of complexity in the processing of 2-argument verbs with two PPs and it warrants further research.

To conclude, this study showed that the visual context presented prior to a reading task can help the parser circumvent the garden-path depending on the lexical properties of the verbs. That is to say, the verbs’ subcategorization information was found to be the factor determining the extent to which context can guide initial parsing decisions. When the argument structure of the verb instigated a bias for a prepositional phrase, the parser tended to attach that prepositional phrase to the verb (i.e., *high attachment*), and this preference was so strong that it could not initially be overridden by the contextual cues. However, when the verb’s argument structure did not call for a prepositional phrase, the contextual cues could successfully guide the parser toward the intended interpretation from the earliest stages of analysis. This finding underscores the importance of taking into account the lexical information of the words while studying attachment preferences.

## Data availability statement

The raw data supporting the conclusions of this article will be made available by the authors, without undue reservation.

## Ethics statement

The studies involving human participants were reviewed and approved by the Research Ethics Committee (CETO) of the Faculty of Arts, University of Groningen. The patients/participants provided their written informed consent to participate in this study.

## Author contributions

SS, RB, and SP contributed to conception and design of the study. SS and SA did the statistical analysis. SS wrote the first draft of the manuscript. All authors contributed to the article and approved the submitted version.

## Funding

SS was supported by doctoral grants from University of Groningen and Macquarie University under the IDEALAB joint doctoral programme [International Macquarie University Research Excellence Scholarship Allocation No. 2020009]. SA declares funding support from the European Union’s Horizon 2020 research and innovation programme under the Marie Skłodowska-Curie [grant agreement no. 838602].

## Conflict of interest

The authors declare that the research was conducted in the absence of any commercial or financial relationships that could be construed as a potential conflict of interest.

The reviewer PV declared a shared parent affiliation with the authors SP, RB, and SS to the handling editor at the time of review.

## Publisher’s note

All claims expressed in this article are solely those of the authors and do not necessarily represent those of their affiliated organizations, or those of the publisher, the editors and the reviewers. Any product that may be evaluated in this article, or claim that may be made by its manufacturer, is not guaranteed or endorsed by the publisher.

## Footnotes

^1^We opted for high attachment as the preferred structure based on the study by Tanenhaus et al. (1995) on double PP structures, but also based on the work on single PP attachment ambiguity (e.g., Frazier and Rayner, 1982; Ferreira and Clifton, 1986). We are aware that this is still a controversial issue and that the debate on which one is the default option is still ongoing and may depend on a number of factors, such as the language. However, what does not seem to be controversial is that there is indeed a preferred interpretation, which is what we exploited in this study. Our goal was not to establish which interpretation is default, but rather whether we can alter the parsing preference (no difference in reaction times) or elicit reanalysis (delay for the dispreferred structure).

^2^ https://www.vyond.com

^3^However, unlike Britt (1994), we found this effect when we compared 2-Argument and 3-Argument structures. The optional and obligatory nature of the PP in 3-Argument structures in this study did not affect processing. Indeed, we added Verb Type (i.e., obligatory vs. optional) as an exploratory fixed effect and it did not improve the model fit. This distinction in the findings might be due to using different types of contexts. That is, Britt provided contextual cues in the form of extrasentential information by presenting a paragraph before the ambiguous sentence whereas we used visual non-linguistic cues.
