# Corrigendum: Does a walk-through video help the parser down the garden-path? A visually enhanced self-paced reading study in Dutch

**DOI:** 10.3389/fpsyg.2023.1151887

**Published:** 2023-02-20

**Authors:** Sara Shoghi, Seçkin Arslan, Roelien Bastiaanse, Srdan Popov

**Affiliations:** ^1^International Doctorate in Experimental Approaches to Language and Brain (IDEALAB), University of Groningen, Netherlands/University of Newcastle, United Kingdom/University of Potsdam, Germany and Macquarie University, Sydney, NSW, Australia; ^2^Center for Language and Cognition Groningen (CLCG), University of Groningen, Groningen, Netherlands; ^3^CNRS, BCL, Université Côte d'Azur, Nice, France; ^4^Department of Neurosurgery, University Medical Center Groningen, Groningen, Netherlands

**Keywords:** syntactic ambiguity resolution, self-paced reading, visual context, sentence processing, PP attachment, written language comprehension

In the published article, there was an error in Figure 1. The figure included two panels, which were placed incorrectly. Panel A should have depicted the images that were visible at the bottom of the figure, while panel B should have depicted the images that were visible at the top. The corrected Figure 1 and its caption appear below.

**Figure 1:**
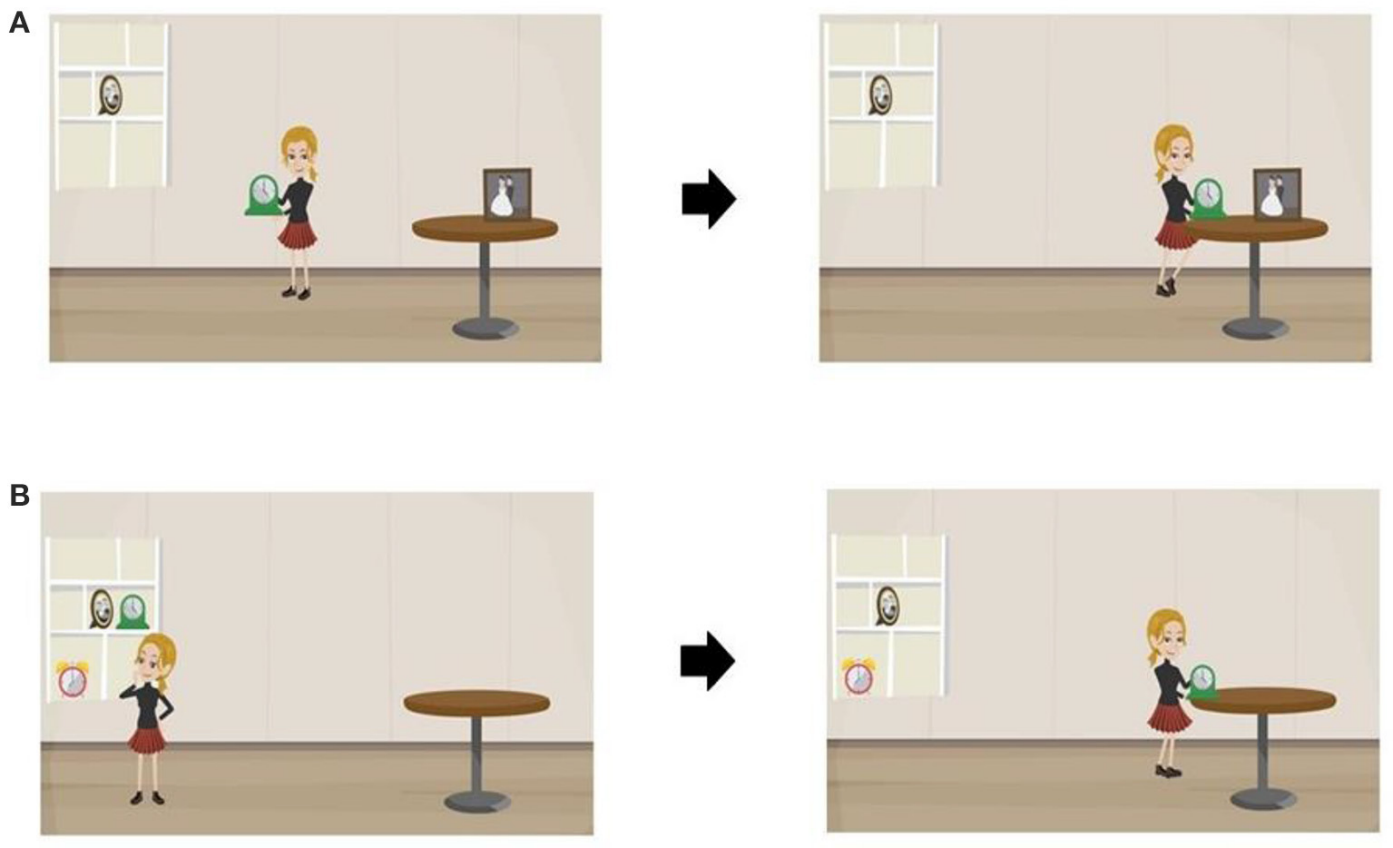
Parts of the animations representing two different attachment types for the sentence “Ze zet de klok naast de foto op de tafel” (She puts the clock next to the photo on the table): **(A)** High-Attachment interpretation. **(B)** Low-Attachment interpretation.

The authors apologize for this error and state that this does not change the scientific conclusions of the article in any way. The original article has been updated.

