# Supplementary material for: Does a walk-through video help the parser down the garden-path? A visually enhanced self-paced reading study in Dutch
Source: Front Psychol. 2022 Dec 21;13:1009265. doi: 10.3389/fpsyg.2022.1009265 (PMC9851380; doi:10.3389/fpsyg.2022.1009265)
Supplement: Supplementary file 1 [file Table_1.docx]

**Appendix 1**

*Output of the Global Mixed-effects Log-transformed RT Model (outliers excluded) for the whole ambiguous sentence plus the spillover region and final word.*

| **Fixed effects** | **Estimate** | **SE** | ***t* value** | ***p* value** |
| --- | --- | --- | --- | --- |
| *Word 1:* ***Ze*** *zet de klok naast de foto op de tafel [spillover region] [Final word].* | | | | |
| (Intercept) | 6.177 | 0.050 | 123.048 | <2e-16 *** |
| Attachment (NP) | -0.020 | 0.010 | -1.974 | 0.048 * |
| Argument (3) | -0.035 | 0.027 | -1.302 | 0.196 |
| Word length | -0.019 | 0.027 | -0.719 | 0.474 |
| Att:Arg | -0.020 | 0.021 | -0.952 | 0.341 |
| *Word 2: Ze* ***zet*** *de klok naast de foto op de tafel [spillover region] [Final word].* | | | | |
| (Intercept) | 5.633 | 0.037 | 148.413 | <2e-16 *** |
| Attachment (NP) | -0.004 | 0.006 | -0.728 | 0.466 |
| Argument (3) | -0.011 | 0.023 | -0.502 | 0.617 |
| Word length | 0.011 | 0.005 | 2.115 | 0.036 * |
| Att:Arg | -0.002 | 0.013 | -0.202 | 0.839 |
| *Word 3: Ze zet* ***de*** *klok naast de foto op de tafel [spillover region] [Final word].* | | | | |
| (Intercept) | 5.609 | 0.049 | 112.749 | <2e-16 *** |
| Attachment (NP) | 0.003 | 0.005 | 0.686 | 0.493 |
| Argument (3) | -0.028 | 0.021 | -1.325 | 0.189 |
| Word length | -0.001 | 0.023 | -0.070 | 0.944 |
| Att:Arg | -0.002 | 0.011 | -0.252 | 0.801 |
| *Word 4: Ze zet de* ***klok*** *naast de foto op de tafel [spillover region] [Final word].* | | | | |
| (Intercept) | 5.633 | 0.037 | 148.413 | <2e-16 *** |
| Attachment (NP) | -0.004 | 0.006 | -0.728 | 0.466 |
| Argument (3) | -0.011 | 0.023 | -0.502 | 0.617 |
| Word length | 0.011 | 0.005 | 2.115 | 0.0369 * |
| Att:Arg | -0.002 | 0.013 | -0.202 | 0.839 |
| *Word 5: Ze zet de klok* ***naast*** *de foto op de tafel [spillover region] [Final word].* | | | | |
| (Intercept) | 5.692 | 0.037 | 151.241 | <2e-16 *** |
| Attachment (NP) | -0.008 | 0.006 | -1.235 | 0.216 |
| Argument (3) | -0.025 | 0.023 | -1.061 | 0.291 |
| Word length | 0.022 | 0.008 | 2.679 | 0.008** |
| Att:Arg | -0.012 | 0.012 | -0.968 | 0.333 |
| *Word 6: Ze zet de klok naast* ***de*** *foto op de tafel [spillover region] [Final word].* | | | | |
| (Intercept) | 5.635 | 0.059 | 95.418 | <2e-16 *** |
| Attachment (NP) | -0.005 | 0.005 | -0.955 | 0.340 |
| Argument (3) | -0.024 | 0.024 | -1.011 | 0.315 |
| Word length | -0.008 | 0.028 | -0.300 | 0.765 |
| Att:Arg | -0.013 | 0.011 | -1.172 | 0.241 |
| *Word 7: Ze zet de klok naast de* ***foto*** *op de tafel [spillover region] [Final word].* | | | | |
| (Intercept) | 5.687 | 0.041 | 138.059 | <2e-16 *** |
| Attachment (NP) | 0.007 | 0.007 | 1.055 | 0.291 |
| Argument (3) | -0.024 | 0.028 | -0.845 | 0.401 |
| Word length | 0.007 | 0.007 | 0.968 | 0.336 |
| Att:Arg | -0.011 | 0.014 | -0.820 | 0.412 |
| *Word 8: Ze zet de klok naast de foto* ***op*** *de tafel [spillover region] [Final word].* | | | | |
| (Intercept) | 5.722 | 0.038 | 147.625 | <2e-16 *** |
| Attachment (NP) | 0.010 | 0.006 | 1.503 | 0.132 |
| Argument (3) | -0.020 | 0.024 | -0.835 | 0.406 |
| Word length | -0.021 | 0.009 | -2.371 | 0.019 * |
| Att:Arg | 0.030 | 0.013 | 2.191 | 0.028 * |
| *Word 9: Ze zet de klok naast de foto op* ***de*** *tafel [spillover region] [Final word].* | | | | |
| (Intercept) | 5.712 | 0.051 | 111.190 | <2e-16 *** |
| Attachment (NP) | 0.025 | 0.006 | 4.258 | 0.000 *** |
| Argument (3) | -0.015 | 0.022 | -0.668 | 0.506 |
| Word length | 0.017 | 0.024 | 0.705 | 0.483 |
| Att:Arg | 0.011 | 0.012 | 0.937 | 0.349 |
| *Word 10: Ze zet de klok naast de foto op de* ***tafel*** *[spillover region] [Final word].* | | | | |
| (Intercept) | 5.701 | 0.041 | 136.397 | <2e-16 *** |
| Attachment (NP) | 0.022 | 0.007 | 2.992 | 0.002 ** |
| Argument (3) | -0.020 | 0.026 | -0.774 | 0.441 |
| Word length | 0.020 | 0.008 | 2.379 | 0.019 * |
| Att:Arg | 0.002 | 0.014 | 0.168 | 0.866 |
| *Word 11: Ze zet de klok naast de foto op de tafel* ***[spillover region]*** *[Final word].* | | | | |
| (Intercept) | 5.709 | 0.051 | 111.393 | <2e-16 *** |
| Attachment (NP) | 0.015 | 0.006 | 2.226 | 0.0261 * |
| Argument (3) | -0.005 | 0.024 | -0.241 | 0.810 |
| Word length | -0.024 | 0.019 | -1.289 | 0.201 |
| Att:Arg | 0.008 | 0.013 | 0.586 | 0.558 |
| *Final Word: Ze zet de klok naast de foto op de tafel [spillover region]* ***[Final word]****.* | | | | |
| (Intercept) | 6.062 | 0.051 | 118.000 | <2e-16 *** |
| Attachment (NP) | 0.013 | 0.009 | 1.369 | 0.171 |
| Argument (3) | -0.003 | 0.030 | -0.124 | 0.901 |
| Word length | 0.002 | 0.009 | 0.325 | 0.746 |
| Att:Arg | 0.021 | 0.019 | 1.087 | 0.277 |

**Note.** Attachment Type and Argument Structure are abbreviated as Att and Arg in the rows representing an interaction.
