# Supplementary material for: Does a walk-through video help the parser down the garden-path? A visually enhanced self-paced reading study in Dutch
Source: Front Psychol. 2022 Dec 21;13:1009265. doi: 10.3389/fpsyg.2022.1009265 (PMC9851380; doi:10.3389/fpsyg.2022.1009265)
Supplement: Supplementary file 2 [file Table_2.docx]

**Appendix 2**

*Output of the Mixed-effects Log-transformed RT Model (outliers excluded) for 2-Argument Verbs and 3-Argument Verbs in Critical Regions (Region 1: the noun preceding PP2, Region 2: the preposition in the PP2, Region 3: the article in the PP2, Region 4: the noun in PP2, Region 5: the spillover region, Region 6: the final word)*

| **Argument Str.** |  | **2AV** | | | |  | **3AV** | | | |
| --- | --- | --- | --- | --- | --- | --- | --- | --- | --- | --- |
| **Fixed effects** |  | **Estimate** | **SE** | **t value** | **p value** |  | **Estimate** | **SE** | **t value** | **p value** |
| *Region 1* | | | | | | | | | | |
| (Intercept) |  | 5.703 | 0.047 | 121.301 | .000 *** |  | 5.708 | 0.043 | 130.582 | .000 *** |
| Attachment (L) |  | 0.014 | 0.012 | 1.126 | 0.2603 |  | 0.006 | 0.012 | 0.534 | 0.594 |
| Word length |  | 0.022 | 0.011 | 2.027 | 0.049 * |  | -0.007 | 0.009 | -0.734 | 0.467 |
| *Region 2* | | | | | | | | | | |
| (Intercept) |  | 5.751 | 0.039 | 146.807 | .000 *** |  | 5.731 | 0.041 | 137.582 | .000 *** |
| Attachment (L) |  | -0.003 | 0.012 | -0.273 | .785 |  | 0.033 | 0.012 | 2.707 | 0.006 ** |
| Word length |  | -0.017 | 0.015 | -1.154 | .255 |  | -0.018 | 0.009 | -1.916 | 0.061 |
| *Region 3* | | | | | | | | | | |
| (Intercept) |  | 5.746 | 0.071 | 80.899 | .000 *** |  | 5.737 | 0.058 | 97.410 | .000 *** |
| Attachment (L) |  | 0.017 | 0.010 | 1.634 | .103 |  | 0.040 | 0.011 | 3.626 | .000 *** |
| Word length |  | 0.023 | 0.039 | 0.597 | .554 |  | 0.027 | 0.032 | 0.852 | .399 |
| *Region 4* | | | | | | | | | | |
| (Intercept) |  | 5.732 | 0.046 | 122.840 | .000 *** |  | 5.726 | 0.045 | 124.570 | .000 *** |
| Attachment (L) |  | 0.020 | 0.014 | 1.398 | .1623 |  | 0.025 | 0.013 | 1.865 | .062 |
| Word length |  | 0.030 | 0.013 | 2.241 | .03 * |  | 0.014 | 0.011 | 1.236 | .224 |
| *Region 5* | | | | | | | | | | |
| (Intercept) |  | 5.733 | 0.065 | 87.834 | .000 *** |  | 5.747 | 0.054 | 106.076 | .000 *** |
| Attachment (L) |  | 0.023 | 0.012 | 1.836 | .066 |  | 0.029 | 0.014 | 2.050 | .04 * |
| Word length |  | -0.021 | 0.032 | -0.677 | .5026 |  | -0.017 | 0.024 | -0.715 | .478 |
| *Region 6* | | | | | | | | | | |
| (Intercept) |  | 6.084 | 0.054 | 111.332 | .000 *** |  | 6.080 | 0.055 | 110.201 | .000 *** |
| Attachment (L) |  | 0.012 | 0.016 | 0.752 | .452 |  | 0.041 | 0.016 | 2.495 | .012 * |
| Word length |  | -0.013 | 0.013 | -0.983 | .33 |  | 0.019 | 0.013 | 1.444 | .156 |

*Note.* * Significant *p* value (<.05); ** Significant *p* value (<.01); *** Significant *p* value (<.001). RTs were natural log transformed. Word length was mean-centered.
